# Magnetic Resonance Imaging Findings of Intrapancreatic Accessory Spleen

**DOI:** 10.5334/jbr-btr.1187

**Published:** 2016-09-21

**Authors:** Damien Le, Ulrike Schierloh, Jean-Paul Van Nieuwenhuyse, Alain Nchimi

**Affiliations:** 1 Centre Hospitalier de Luxembourg, LU

**Keywords:** Spleen, Pancreas, Abnormalities, MRI

A 15-year-old girl was referred for facial hirsutism and obesity. She had been seen five years ago for premature pubarche and otherwise had no previous medical or surgical history. Other physical examination findings were normal. Laboratory tests showed elevation of serum free testosterone and D4-androstenedione. Transabdominal ultrasonography (US) revealed a bilateral excessive ovarian size, numerous small peripheral follicles and increased central stroma, suggesting the diagnosis of polycystic ovary syndrome. However, US also demonstrated a 2.5 x 2.3 cm pancreatic tail mass (asterisk, Figure 1A). Magnetic resonance imaging (MRI) was performed subsequently and confirmed the presence of a mass of the dorsal surface of the pancreatic tail. The mass was mildly hypointense on T1-weighted imaging, and hyperintense on T2-weighted imaging compared to the surrounding pancreas, but was isointense to the spleen on all unenhanced sequences, including Diffusion-weighted imaging (DWI) sequences (with b-values of 50 and 800 sec/mm^2^, Figures 1B and 1C, respectively). It showed however a slightly distinctive enhancement pattern to the spleen on dynamic contrast-enhanced MRI (i.e. slightly hypointense imaging of the spleen on early and late arterial phases and isointense on venous phase) (Figures 1D, 1E and 1F, respectively). The remaining pancreatic tissue was normal and no lymphadenopathy or other pathologic process was found in the abdomen. Altogether, we arrived at the diagnosis of an intrapancreatic accessory spleen (IPAS) and a follow-up (3 months) US confirmed the stability of the lesion.

**Figure 1:**
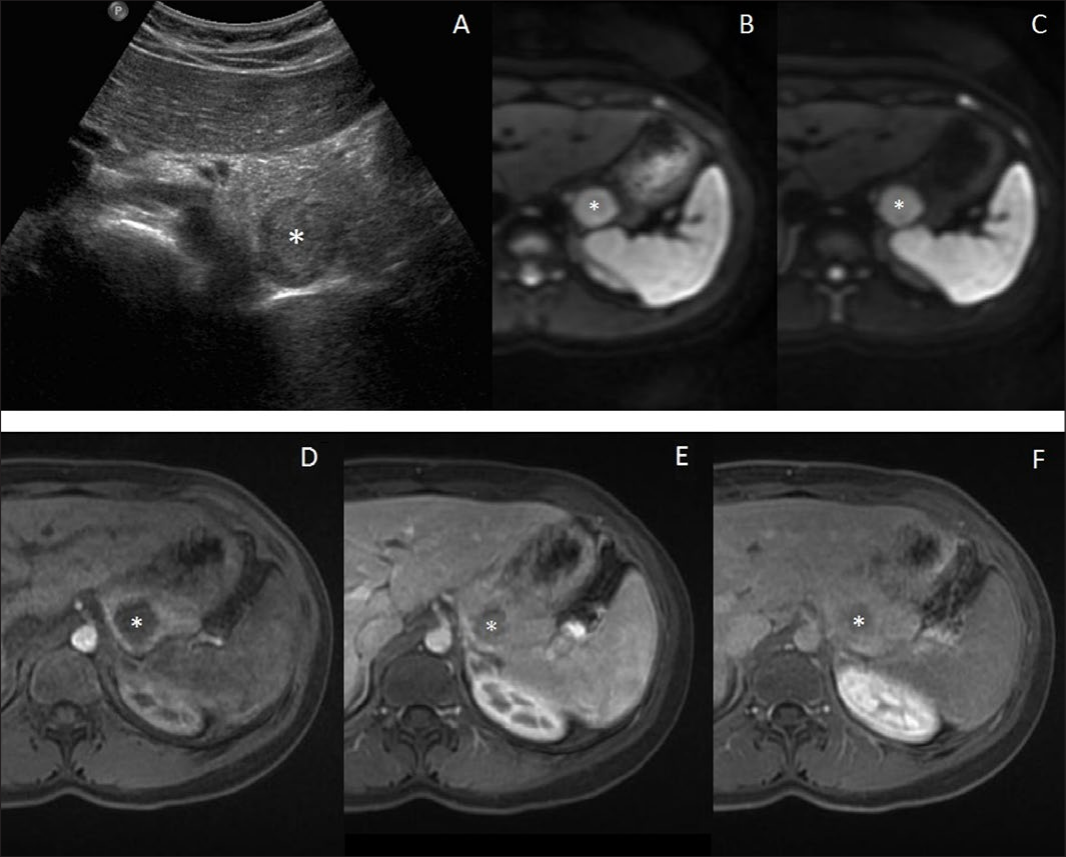


## Comments

IPAS represents a congenital anomaly arising from aberrant splenic embryologic fusion, resulting in the ectopic deposition of normal splenic tissue in the pancreatic parenchyma. The most common location is the tip of the pancreatic tail, usually along its dorsal surface. Typically, IPAS demonstrates similar signal intensities and similar enhancement compared to the spleen on MRI [1]. However, pancreatic neuroendocrine tumor (PNET) and solid pseudopapillary tumor (SPPT) can share imaging characteristics with IPAS on T1- and T2-weighted MRI. Contrast enhancement patterns have been used to distinguish these two malignancies from IPAS. Indeed, PNET often shows a uniform or ring-like enhancement mostly during the early arterial phase, while the typical appearance of SPPT is early, peripheral and heterogeneous enhancement during the arterial phase with progressive but heterogeneous filling during the portal venous and late phases [2]. The enhancement of SPPT is lower than the adjacent normal pancreas on all phases [2]. Nevertheless, some IPAS may exhibit atypical enhancement as in the present case, as the enhancement was delayed on arterial phases. In such cases it is recommended to consider DWI findings to sort the differential diagnosis [1]. The signal similarity to the spleen on all b-value sequences outclasses all other morphologic and enhancement findings for the diagnosis of IPAS.

In conclusion, a combined review of MRI with morphologic sequences, dynamic enhancement and DWI can be useful to noninvasively characterize intrapancreatic accessory spleen and differentiate it from other solid pancreatic masses.
